# Validation of the Spanish version of the Psychological Flexibility Sports Scale (PFSS)

**DOI:** 10.3389/fpsyg.2025.1731006

**Published:** 2026-02-04

**Authors:** Alejandro Garcia-Pardina, Oscar A. Belloso, Alberto Bellido-Esteban, Almudena Montalvo-Pérez, Lidia Budziszewska

**Affiliations:** 1 Department of Psychology, Faculty of Biomedical and Health Sciences, Universidad Europea de Madrid, Madrid, Spain; 2 Department of Real Madrid Graduate School, Faculty of Medicine, Health and Sports, Universidad Europea de Madrid, Madrid, Spain

**Keywords:** sport psychology, psychometric properties, reliability, validity, confirmatory factor analysis

## Abstract

**Introduction:**

Psychological flexibility has been conceptualized as the capacity to relate to internal experiences, maintain focus on the present moment, thereby allowing individuals to act according to the context in pursuit of their goals and values. It is a psychological construct related to mental health, where there is an increasing body of research that highlights its importance in athletic performance. The Psychological Flexibility Sports Scale (PFSS) is the first psychological flexibility scale specifically designed for use in sports. It has only been validated in a limited number of cultural contexts. Concerning this, the study aims to translate the PFSS into Spanish (ES-PFSS), thereby analyzing and identifying the psychometric properties of the ES-PFSS among native Spanish athletes or those with medium to high proficiency in Spanish.

**Methods:**

The sample comprised 146 participants who practiced both team-based and individual sports. (Age 33.95 ± 11.69 years, 63.014% male). Statistical analysis was performed for reliability (internal consistency and temporal stability), construct validity, and criterion validity.

**Results and discussion:**

The scale was confirmed to be unidimensional through confirmatory factor analysis. Significant results were found for the evaluation of construct validity. The ES-PFSS demonstrated solid criterion validity related to other constructs of psychological flexibility and perceived athletic performance. The reliability study proved to be significant, with excellent internal consistency (Cronbach’s *α* = 0.937). The scale showed low temporal stability (ICC = 0.378).

## 1 Introduction

Athletes face numerous psychological barriers in the pursuit of optimal athletic performance, within which intrusive thoughts, emotions, and behavioral predispositions are considered essential and common hindrances, creating obstacles to obtaining optimal performance. Therefore, it is crucial to identify and conceptualize psychological variables that play a role in athletic performance. Psychological flexibility is the ability to accept and be willing to experience unwanted private events to achieve personal values and goals (Hayes et al., 1996, 2006). It was developed as a psychological variable within the context of Acceptance and Commitment Therapy (ACT), including processes like acceptance and values to change the way patients react to anxiety, depression, and stress (Hayes et al., 2004). Psychological flexibility is a construct that is interwoven into the ACT framework initially described by Hayes et al. (1999), where he proposed a model based on six processes: acceptance, contacting the present moment, cognitive diffusion, self-as-context, engaged action, and values. According to the ACT model, the value-based opportunities present in each context require individuals to adjust how much they rely on immediate contingencies versus their internal experiences, whether those experiences are unwanted, wanted or neutral. In contrast, psychological inflexibility (PI) describes a rigid behavioral style where individuals act primarily in response to momentary internal experiences rather than aligning with their chosen values (Bond et al., 2011). It consists of a rigid dominance of psychological reactions over chosen values and their committed actions, which usually occurs when the individual merges with self-evaluations and thoughts that prevent contact with experience, often increasing psychological distress. To highlight ACT’s emphasis on flexibility, its underlying model has been referred to as psychological flexibility or simply flexibility (Hayes et al., 1999, 2006).

In sport contexts, where athletes are required to meet performance demands, adapt and overcome competitive pressures and face unique contextual factors inherent to athletic settings, psychological flexibility has been recognized as a key component that enhances adaptive functioning and optimal performance. Psychological flexibility in athletes is defined by openness to aversive experiences without efforts to change their occurrence or specific form (emotions, thoughts, memories or physiological responses) (Lundgren et al., 2018). That is, their committed behavior is directed by both athletic and personal values across training, competition, and other areas such as social, interpersonal, and relational contexts. The athlete’s actions are in touch with the present moment and aware of the events unfolding (internal and external) to be open and consciously seek the best actions that lead to effective performance (e.g., distancing from intrusive thoughts, adjustment and adaptability of strategies given by coaches and other professionals, emotional regulation, awareness of emotions, etc.) (Lundgren et al., 2018).

Applying psychological interventions such as mindfulness and acceptance has shown a reduction in behavioral issues, emotional distress, and other psychological symptoms, along with improvements in athletic performance (Gross et al., 2018). Mindfulness and acceptance techniques are frequently utilized with athletes and artists to foster well-being and boost performance. As described by Latinjak et al. (2019), throughout the competition process (preparation, competition, and recovery), athletes face the effects of automatic thinking in its three forms: spontaneous, stimulus-independent thinking, and mind wandering. Spontaneous thinking is effortless, however, dependent on relevant cues in each context or task. Stimulus-dependent thinking is also automatic but does not rely on specific task-linked cues. Latinjak et al. (2023) advances the comprehension of automatic thinking and integrates it into a functional, meaning-based model of self-talk, emphasizing context, intention, and personal significance rather than rigid classifications (e.g., positive vs. negative). Self-talk would allow us to acknowledge emotional states and therefore increase control over them. This comprehension of self-talk is close to the mindfulness and acceptance approach.

Lastly, mind-wandering is unrelated to the ongoing activity. Mindfulness-based interventions focus on promoting a change in how an athlete relates to internal experiences, striving to instill a non-judgmental awareness of these thoughts and emotions. Research by Kettunen and Välimäki (2014) aimed at investigating whether a six-week ACT-based psychological coaching intervention would have positive effects on psychological and sports-related factors of floorball players. The intervention influenced athletes’ self-confidence in sports positively and lowered levels of experienced stress; however, the overall results show that the application of ACT did not have significant effects on the subjects ‘being or self-evaluated performance’. Nonetheless, a more recent study by Wang et al. (2023) explored the effects of mindfulness-based interventions (MBIs) on enhancing athletic performance, demonstrating significant improvements in sport performance. In another study, results found that basketball athletes practicing 15 min of mindfulness interventions improved first free throw performance under a stressful setting compared to control conditions (Wolch et al., 2020). Mindfulness- and acceptance-based interventions are mentioned given that they constitute core processes within the ACT theoretical framework, are used to enhance psychological flexibility, and view psychological flexibility as a central component and mechanism of change (Hayes et al., 1999, 2006).

Given that psychological flexibility is considered a central component through which mindfulness- and ACT-based interventions exert their outcomes, it is of importance to evaluate the effectiveness of said interventions which relies on the capacity to accurately and validly measure psychological flexibility within sport-specific contexts. Current psychological flexibility measures may not fully grasp the complexity of sport-specific contexts, considering the performance, competitive and contextual demands, highlighting the need for instruments specifically designed for use in sport. Due to the lack of valid sport-specific measures of psychological flexibility, it is difficult to determine whether mindfulness- and ACT-based interventions effectively strengthen this construct. This limitation hampers meaningful comparison and synthesis of findings across studies and constrains theory-driven progress directed toward ACT-based models within sport contexts. Considering this need, sport-specific instruments have been developed to assess psychological flexibility in athletic settings, providing researchers with tools that account for the specific demands of sport.

Based on ACT therapy and current sport psychology research, psychological flexibility is strongly linked to indicators of psychological well-being, distress, and performance-related perceptions in sport contexts. Higher psychological flexibility has been associated with lower levels of psychological distress as well as with greater life satisfaction and adaptive functioning. Furthermore, psychological flexibility is theoretically supported with acceptance- and values-based processes evaluated by general measures of flexibility. For that reason, analyzing associations between psychological flexibility in sport and related constructs such as depression, anxiety, stress, life satisfaction, and general psychological flexibility provides a theoretical groundwork for evaluating criterion validity. In addition to mental health outcomes, psychological flexibility has been suggested as an important ability that ensures effective performance under competitive pressure. Athletes able to remain present, accept private experiences, and act in line with their values may be more capable of sustaining optimal performance. Therefore, it is hypothesized that (1) ES-PFSS scores will show a significant positive correlation with other measures of psychological flexibility and with other constructs of psychological health (depression, anxiety and stress), and negatively with quality of life; (2) participants with lower ES-PFSS scores (indicating greater psychological flexibility) will self-report better sports performance at the competitive level.

While the application of mindfulness and acceptance interventions in sports is increasing, ongoing development and research of instruments are becoming more critical to comprehend psychological flexibility in performance so that researchers and practitioners can evaluate training procedures as well as the impact that therapies and processes linked to gaining psychological flexibility have. Although it is recognized that psychological flexibility plays a critical role in optimal athletic performance, the current available literature does not provide a complete representation, and its relationship is only recently being explored.

In view that there is growing evidence that mental health and optimal performance are influenced by psychological flexibility, i.e., how people experience thoughts and feelings, it is of great interest to look for ways to measure this construct in the sports setting. Previously, psychological flexibility was explored using the AAQ-II scale (Acceptance and Action Questionnaire-version II), an example of a study conducted by Zhang et al. (2014) where the psychometric properties of the instrument were explored in elite athletes in China. Currently, a psychometrically adapted measure of psychological flexibility in the athletic population called the Psychological Flexibility in Sport Scale (PFSS) has been found. The instrument was created by Johles et al. (2020) and consists of a general measure of psychological flexibility, which allows for the assessment of that construct across a broad spectrum of athletes. The scale was tested in a sample of 152 Swedish participants and demonstrated one-dimensionality (seven items), finding acceptable psychometric properties.

Nevertheless, the availability of translated and culturally adapted psychometric scales is essential for further advancement and innovation in psychological science. In line with Acceptance and Commitment Theory (ACT), psychological flexibility differs depending on cultural context (Hayes et al., 1999). Recently, an Iranian study tested the P-PFSS (an adaptation to Persian) and found that the factorial structure of this version differed slightly (included two substantial correlated residuals) from that of the Swedish version, emphasizing the influence of culture on psychological flexibility (Badinlou et al., 2022). While the scale specifically measures psychological flexibility in sports, it has been evaluated in a handful of contexts, notably the study in Sweden by Johles et al. (2020), as well as in Australia (Rutherford, 2021). Therefore, there is a need to translate and validate scales in multiple athlete populations, thus improving their practical utility and promoting a greater insight into the psychometric performance of revised scale versions among populations that have yet to be investigated.

Accordingly, the aim of the present study is to translate and psychometrically evaluate the Psychological Flexibility in Sport Scale (ES-PFSS) for Spanish-speaking athletes in Spain. The target population includes athletes competing at regional, national, or international levels, in either individual or team sports, who follow structured or periodized training programs and are actively developing the skills required for high-level performance. Participants are also required to have at least an intermediate to high level of Spanish proficiency. The study specifically seeks to analyze the factorial structure and assess the reliability of the scale. Furthermore, it aims to evaluate the psychometric properties of the ES-PFSS in terms of reliability (internal consistency and temporal stability), construct validity and criterion validity in the population of Spanish athletes.

## 2 Method

### 2.1 Ethics statement

Ethical approval was granted by the European University of Madrid by the European University Ethics Committee (CEI-UE 2024-576). The participants provided informed consent by completing the form. This form was presented at the beginning of the survey, a prerequisite for proceeding to subsequent sections. All participants received the informed consent form in digital format and were granted access to digital copies throughout the study. Participants were informed of their right to withdraw from the study at any point prior to the closure of the survey. No external sporting body or organization or research team had access to the data. Participation was voluntary and no reward or compensation was provided for participants. All data generated or analyzed during this study are available from the corresponding author on reasonable request.

### 2.2 Participants

Recruitment was carried out via convenience sampling, involving the cooperation of professionals, organizations, and personal contacts of the authors. Using McKay et al.’s (2022) classification of “elite” athletes, we chose those at levels two (Trained/Developmental Level), three (Trained/National Level), and four (Elite/International Level). Athletes who comply with periodized training three times per week, who identify with a single sport, and who compete locally, nationally, or internationally, meet the standards for being in level two, three, four, and five. The participant is adapted to this classification using questions in the sociodemographic data section, such as level of competition, type of contract the athlete has, work apart from the sport dedication, years practicing the sport, and time dedicated to the competitive level of the discipline. With that said, the following inclusion criteria were chosen: (1) Medium to high proficiency or understanding of Spanish, (2) Participants compete at a national or international level (team or individual sport), in an amateur, semi-professional or professional level, (3) Perform structured or periodized training weekly and develop the necessary skills to perform their sport, 18 years or older. In the sociodemographic data section, there are two questions designed to classify the participants according to the criteria, where the participants are asked about the time dedicated to sport.

The study included a sample of 146 participants, with an average age of 33.95 years (± 11.69 years). The sociodemographic data is presented in Table 1. Most of the participants who have a higher education (*n* = 86) play in individual sports (*n* = 100), where 14.384% compete at the professional level. In relation to McKay et al.’s (2022) athlete classification, the sample corresponds to Levels 2–4, which includes trained/developmental, trained/national, and elite/international athletes. The proportion of professional athletes in the sample (14.38%) is aligned with Level 4 (Elite/International), whereas non-professional and semi-professional athletes competing at regional or national levels correspond with Levels 2 and 3. Men reported a mean of 16.69 ± 10.26 years of sport practice and competitive experience, whereas women reported a mean of 9.47 ± 7.96 years.

**Table 1:** Sociodemographic descriptive data.

Variable	*n*	Percentage
Gender		
Masculine	92	63.01
Feminine	53	36.30
Education		
Compulsory secondary education (ESO)	4	2.74
Vocational education	24	16.44
Baccalaureate	32	21.92
Superior education	86	58.90
Competition level		
Amateur	82	56.16
Semi-professional	43	29.45
Professional	21	14.38
Type of contract		
No contract	103	70.55
Unpaid contract	24	16.44
Paid contract	19	13.01

### 2.3 Procedures

The translation process consisted of three steps, (following the standards and regulations of the International Test Use Guidelines; ITC). First, the scale was given to bilingual clinical psychologists, native speakers in Spain. Each independently developed a translation of the instructions, items and Likert scales. The two resulting versions were then compared and integrated for the development of the final version. Second, the Spanish version was again translated into English by two English-speaking translators, one British and one American, both experts in clinical psychology and bilingual. Third, the original English scale and both translated versions are checked by the two authors, highlighting no significant differences. Based on this translation process, we have chosen to rename the scale as ES-PFSS and use this name when administering the scale to participants.

An anonymous survey was developed in Google Forms that included informed consent, and the relevant questionnaires were divided into sections. The sections are sociodemographic data, information related to the Borg CR100, quality of life assessment, psychological health, psychological flexibility, and the scale under study, administered in that order. The questionnaire was distributed in several ways, mainly sent online and via QR codes. About the latter tool, a poster with the corresponding QR code was designed and exhibited in different sports associations in Madrid, social media, and at the European University of Madrid.

### 2.4 Instruments and measures

#### 2.4.1 Sociodemographic data

The background questionnaire gathered demographic information (age, gender, education, self-assessed economic situation, marital status, level of competition: regional, national, international; and type of sport: individual or team). Participants were additionally surveyed regarding the age at which they began training in their primary sport, the type of sport practiced and their total years of sports experience. Spanish proficiency was verified by asking the participants to indicate whether they had a medium to high level of Spanish.

#### 2.4.2 Variables related to athletics

Measuring perceptual domains in sports is more challenging than measuring physiological or performance domains, as it is by nature subjective and private. Following the assessment model of sport-related variables by Johles et al. (2020), the Borg CR100 scale is a visual assessment instrument that measures perceived performance in a scale that ranges from 0% to 100% (Borg and Borg, 2002). It is similarly successfully employed to rate the performance of divers (Borg and Love, 2018), golf players (Molander et al., 2013), and in exploring training load in elite soccer players (Fanchini et al., 2016). This scale allows real-time assessment, providing a fast and practical method to obtain performance information. This being a percentage scale filled in with descriptors of such numerical values, e.g., 90% being “extremely strong,” 100% being maximal, 20% weak, 10% very weak, etc. However, in previous studies for the evaluation and validation of the PFSS scale, such an instrument has been used with both the athlete and the coach in mind (Badinlou et al., 2022). Not having access to the coaches of the participants, as well as not having access to the information of the participants at different moments of the competition, the Borg CR100 has been adapted so that the athletes only answer “Yes” or “No” based on the outcome of their competition or preparation phase.

#### 2.4.3 Variables related to psychological health

The DASS-21 (Depression, Anxiety and Stress Scale) was administered. The scale measures these constructs in the last week, being considered a reference tool and of high relevance in clinical research. It consists of 21 items with a 4-point Likert scale from 0 = “It has not happened to me at all” to 3 = “It has happened to me a lot, or most of the time” (Lovibond and Lovibond, 1995). The DASS-21 and its subscales have demonstrated acceptable internal consistency, temporal stability, and convergent validity (Crawford and Henry, 2003). Studies have been carried out about the construct validity and internal consistency of the DASS-21 in Spain, showing better adjustment indexes in comparison with the rest of the alternative models, presenting to be invariant, reliable, and an adequate instrument in the Spanish population (Fonseca-Pedrero et al., 2010). The Cronbach’s alphas in the current sample were: 0.91 (Depression), 0.86 (Anxiety), and 0.89 (Stress).

#### 2.4.4 Quality of life

The SWLS (Satisfaction with Life Scale) was used to measure quality of life. It is a five-item scale that measures overall satisfaction with life. The items are rated on a 7-point Likert scale, from 1 = “I Strongly Disagree” to 7 = “I Strongly Agree” (Diener et al., 1985). In its Spanish version (Vázquez et al., 2013), it showed acceptable test–retest reliability internal consistency and criterion validity. The Cronbach’s alpha in the current sample was 0.80.

#### 2.4.5 Psychological flexibility scale

The AAQ-II (Acceptance and Action Questionnaire II; Bond et al., 2011) was used to assess psychological flexibility. It is considered the reference psychometric tool in the evidence-based study of acceptance, engagement, and mindfulness. Also, it is frequently employed in sport and high-performance contexts, being administered in research focused on rehabilitation among athletes (Zhang et al., 2014) and in the development of the psychometric scale: Psychological Flexibility in Sport Scale (PFSS) (Johles et al., 2020). It is a seven-item measure rated on a seven-point Likert scale ranging from 1 = “Never true” to 7 = “Always true.” Higher scores indicate greater experiential avoidance and immobility, while lower scores reflect greater acceptance and engagement in action. Regarding its psychometric validity and reliability, it presents excellent internal consistency as well as criterion validity by Badinlou et al. (2022). The Cronbach’s alpha in the current sample was 0.941.

#### 2.4.6 Psychological Flexibility Sports Scale (ES-PFSS)

It was designed to assess psychological flexibility in the population of athletes, thus ensuring better understanding of this construct in the context of sport and high-performance activities. It identifies a single factor, psychological flexibility, negatively associated with anxiety and depression (Johles et al., 2020). The scale consists of seven items, each having a seven-point Likert scale from 1 = “Never true” to 7 = “Always true,” where higher scores indicate less psychological flexibility. The PFSS presents excellent internal consistency as well as significant construct, concurrent, and convergent validity (Johles et al., 2020).

The seven items of the Spanish version are as follows: 1. Mis recuerdos y experiencias de fracasos anteriores tienen un impacto negativo en mi rendimiento. 2. Cuando estoy compitiendo no puedo controlar mis nervios y eso afecta negativamente a mi rendimiento. 3. Cuando compito mis pensamientos debilitan/perjudican mi rendimiento. 4. Cuando compito mis sentimientos debilitan/perjudican mi rendimiento. 5. Parece que la mayoría de los atletas pueden manejar sus sentimientos mejor que yo cuando están compitiendo. 6. La ansiedad por rendimiento perjudica mi rendimiento durante las competiciones. 7. La preocupación hace que mi rendimiento empeore cuando estoy compitiendo.

### 2.5 Data analysis

#### 2.5.1 Descriptive statistics and reliability

Descriptive statistics of the sample are reported through relative frequencies of demographic variables (age, gender, marital status, educational level, self-perceived economic status, level of competition: regional, national, international; type of sport: individual, team; age of training onset, and years of athletic experience). Additionally, item-level statistics (means and standard deviations) are calculated, and an inter-item correlation matrix is constructed. The reliability of the scale scores is assessed using two approaches: internal consistency and temporal stability. Internal consistency is evaluated using Cronbach’s alpha and McDonald’s omega coefficients, with values equal to or greater than 0.70 considered acceptable, and 0.80 or higher preferred, in line with the original validation study (0.89). Temporal stability is examined using a test–retest method in a convenience subsample over a five-week interval (*n* = 13). The five-week retest interval was selected to reduce potential recall effects while remaining short enough to limit true change in the construct, calculating the Intraclass Correlation Coefficient (ICC) (2,1; two-way random-effects model, absolute agreement, single measurement) with 95% confidence intervals (consistent with COSMIN recommendations; Mokkink et al., 2024). Also, mean-level change across administrations was examined with a paired-samples t-test to evaluate potential systematic shifts between measurements. ICC was preferred over Pearson’s correlation because it reflects reproducibility rather than only linear association. ICC magnitudes were interpreted using commonly cited guidelines (e.g., Koo and Li, 2016; < 0.50 low, 0.50–0.75 moderate, 0.75–0.90 good, > 0.90 excellent).

#### 2.5.2 Construct validity

Construct and criterion validity are also examined. To assess construct validity, a Confirmatory Factor Analysis (CFA) is conducted to test the hypothesized unidimensional structure of the PFSS, following the original version (Johles et al., 2020). Given the response scale includes more than five categories, the Robust Maximum Likelihood (RML) estimation method is applied. Model fit is assessed by calculating the CFI, TLI, RMSEA, and SRMR, considering the following acceptable thresholds: CFI and TLI > 0.90, RMSEA < 0.08, and SRMR < 0.08 (Hu and Bentler, 1999).

When initial model fit indicated potential localized misfit, modification indices were inspected cautiously and only substantively justified adjustments were considered, like the ones suggested in the Persian adaptation (Badinlou et al., 2022) of the scale. Competing CFA models were compared based on overall fit indices and information criteria (AIC, BIC, ECVI). Standardized factor loadings were interpreted following Brown (2015), whereby values of 0.40 or higher are commonly considered salient in CFA. Additionally, composite reliability (CR) and Average Variance Extracted (AVE) were calculated to assess model-based reliability and convergent validity. To examine whether the ES-PFSS operated equivalently across gender, we conducted an exploratory multi-group confirmatory factor analysis (MG-CFA). Model comparisons were evaluated using robust chi-square difference tests and changes in fit indices.

#### 2.5.3 Criterion validity

Criterion validity is analyzed through correlations between ES-PFSS scores and related constructs, including the Depression, Anxiety, and Stress Scale (DASS-21), the Satisfaction with Life Scale (SWLS), and the Acceptance and Action Questionnaire II (AAQ-II). It is hypothesized that ES-PFSS scores will correlate negatively with DASS-21 scores and positively with SWLS and AAQ-II scores. Correlations of 0.3, 0.5, 0.7 are considered low, moderate, and high association, respectively (Safrit and Wood, 1995). Also, criterion validity is assessed using an independent-samples *t*-test to compare participants who did or did not perceive their most recent competition performance as meeting expectations.

#### 2.5.4 Sample size and software

**Figure 1:**
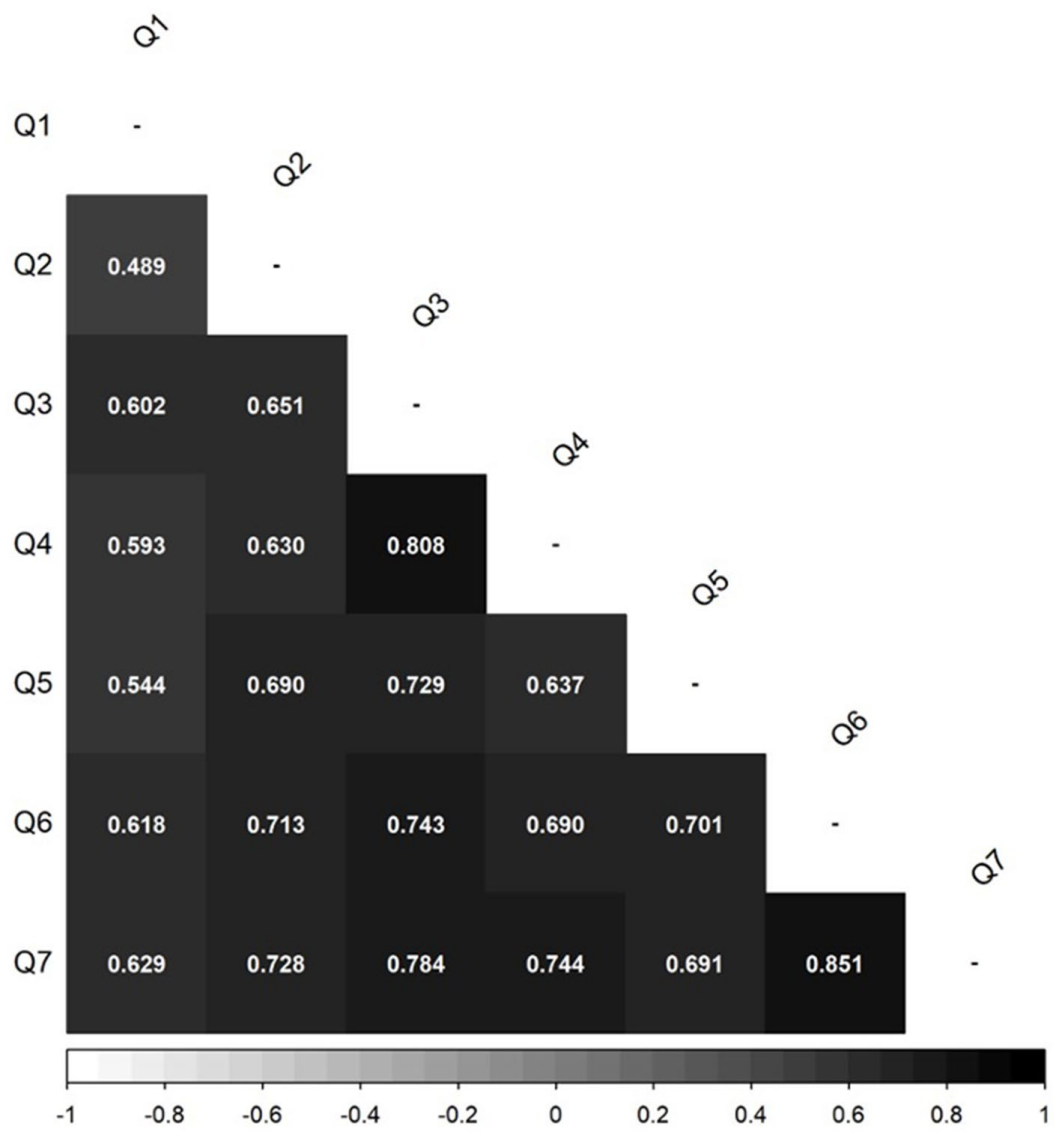
ES-PFSS inter-item Pearson’s correlation matrix.

**Figure 2:**
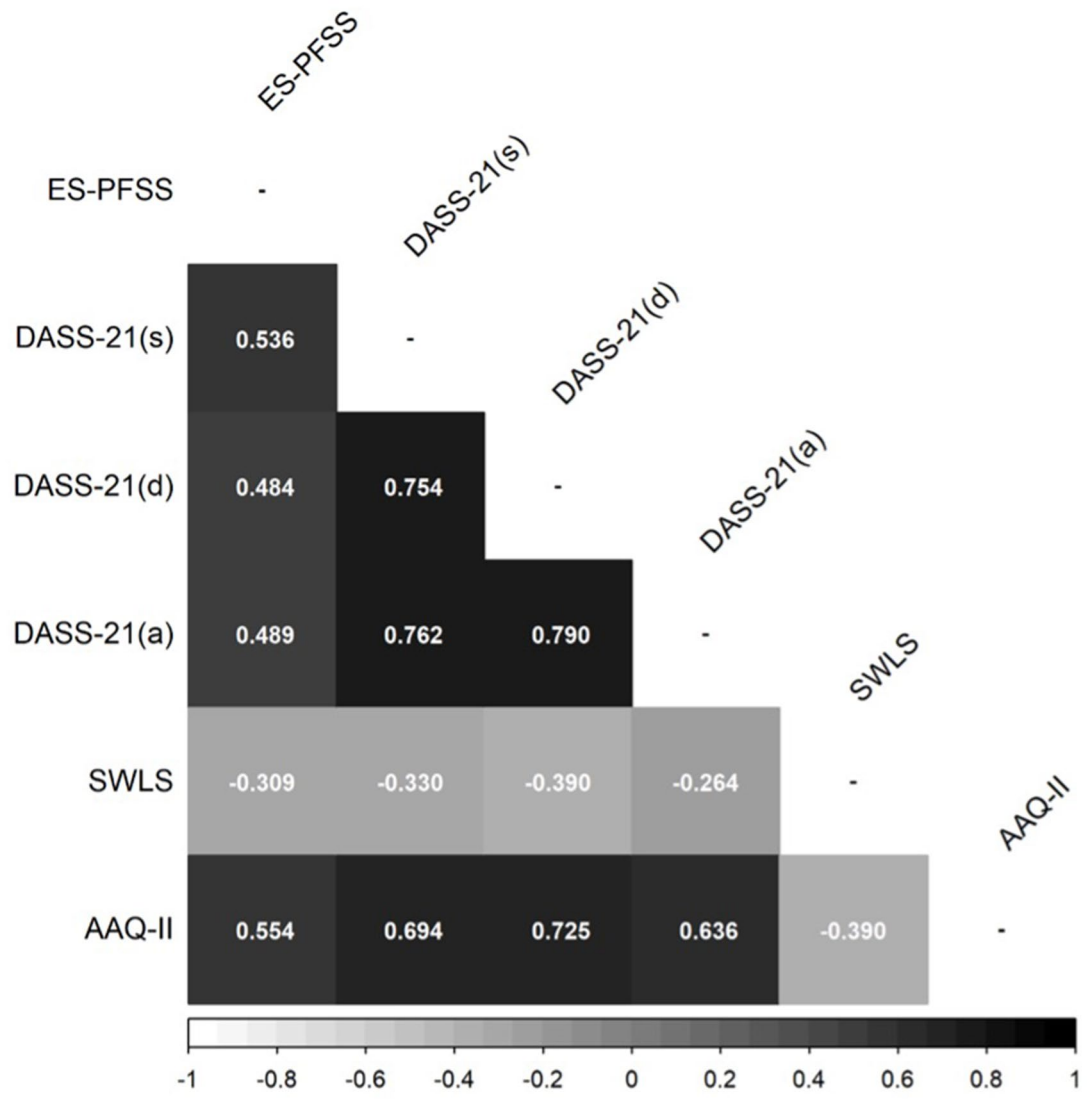
Pearson’s correlation coefficient between ES-PFSS and other scales. ES-PFSS, Psychological Flexibility Sports Scale; DASS-21, Depression (d), Anxiety (a) and Stress (s) Scale; SWLS, Satisfaction with Life Scale; AAQ-II, Acceptance and Action Questionnaire II.

Regarding sample size, the PFSS response scale includes 7 categories. For this reason, responses are treated as continuous variables, based on evidence showing that when Likert scales have five or more categories, treating data as continuous provides estimation precision comparable to ordinal methods (Finney and DiStefano, 2006; Green et al., 1997). Also, given that the hypothesized model implied a single-factor structure with seven items and based on simulation studies on parameter recovery of data that is continuous (Mundfrom et al., 2005), it was determined that sample sizes between 15 and 20 participants per parameter were sufficient for reliable estimation. This is assuming factor loadings between 0.6 and 0.8, as observed in the original validation (Johles et al., 2020). To ensure comparability, a sample size of approximately 150 participants was established, consistent with the original study. The statistical analyses were conducted using JASP software (ver. 0.18.0.3) to calculate descriptive statistics and R software (ver. 4.2.3) to generate the correlation matrices displayed in Figures 1, 2 (corrplot package v. 0.95) and estimating the CFA model (lavaan package v. 0.6-19).

## 3 Results

### 3.1 Descriptive statistics and reliability

First, the averages, standard deviations and ranges of the test scores (SWLS, DASS-21 and AAQ-II) are presented in Table 2. Second when assessing the functioning of the items of the ES-PFSS, we first inspected the inter-item correlation matrix (Figure 1). All correlations were statistically significant at the 95% confidence level and ranged between 0.60 and 0.81, except for the Q1-Q2 pair, which was also statistically significant but presented a value of 0.49. Given that the correlations were large but not excessively close to 1, this suggests evidence of homogeneity: the items share most of their variance while retaining uniqueness in covering different aspects of the construct.

**Table 2:** Descriptive statistics of test scores.

Scale	Average ± SD	Range
SWLS	28.80 ± 4.24	14–35
DASS-21 (d)	3.97 ± 4.34	0–21
DASS-21 (a)	3.65 ± 3.90	0–21
DASS-21(s)	6.34 ± 4.45	0–21
AAQ-II	16.58 ± 9.18	7–49

DASS-21, Depression (d), Anxiety (a) and Stress (s) Scale; SWLS, Satisfaction with Life Scale; AAQ-II, Acceptance and Action Questionnaire II.

In terms of reliability, the overall Cronbach’s *α* is 0.937 and the McDonald’s *ω* is 0.940. Also, their value if a given item were removed ranged from 0.920 to 0.940 (Table 3), meaning that the items are consistent across different measurements, indicating strong internal reliability of the scale. To determine test–retest reliability, a subsample of participants was asked to complete the instrument again after 5 weeks of taking the questionnaire (*n* = 13). The intraclass correlation was ICC (2,1) = 0.378 (95% CI [−0.107, 0.745]) indicating low temporal stability. The paired-samples *t*-test did not indicate a statistically significant mean-level change between the administrations: *t*(12) = 1.961, *p* = 0.073.

**Table 3:** Item descriptive statistics and internal consistency.

ES-PFSS item	Average ± SD	α (if item removed)	ω (if item removed)
Q1	3.103 ± 1.442	0.939	0.940
Q2	2.884 ± 1.417	0.931	0.934
Q3	3.178 ± 1.630	0.922	0.926
Q4	2.932 ± 1.622	0.927	0.931
Q5	3.130 ± 1.645	0.930	0.934
Q6	3.164 ± 1.644	0.923	0.926
Q7	3.130 ± 1.666	0.920	0.923

α, Cronbach’s alpha; ω, McDonald’s omega; ES-PFSS, Spanish Psychological Flexibility Sports Scale; Q, Question; SD, Standard deviation.

### 3.2 Construct validity

For assessing the construct validity, we first estimated the one-factor CFA model (Model 1) based on the original structure proposed by Johles et al. (2020). The resulting fit indices were: Χ^2^(14) = 45.136, *p* < 0.01; CFI = 0.963; TLI = 0.944; RMSEA = 0.123 (90% CI: 0.084–0.165); SRMR = 0.03. Considering the cutoff points suggested by Hu and Bentler (1999), the RMSEA considerably deviated from the consensual criteria. This deviation may be attributed to model misspecification, such as residual covariances that were not accounted for (Saris et al., 2009).

Upon examining the modification indices, the largest value was found for the residual covariance between Q3 and Q4, with an expected change in Χ^2^ of 21.934 and an EPC of 0.344. This adjustment is substantively meaningful, given the nearly identical wording of the two items, differing only in the terms *thoughts* versus *feelings*: Q3 (“Cuando compito mis pensamientos debilitan/perjudican mi rendimiento”; “When I am competing, my thoughts impair my performance”) and Q4 (“Cuando compito mis sentimientos debilitan/perjudican mi rendimiento”; “When I am competing, my feelings impair my performance”). Consequently, we re-estimated the model (Model 2), specifying this as a correlated residual. Model 2 showed a marked improvement in fit: Χ^2^(13) = 23.936, *p* = 0.032; CFI = 0.987; TLI = 0.979; RMSEA = 0.076 (90% CI: 0.022–0.123); SRMR = 0.022. These values align much more closely with the recommended criteria. Information criteria also favored Model 2 over Model 1 (∆AIC = −19.20; ∆BIC = −16.22; ECVI = 0.369 vs. 0.501), supporting the revised specification. The correlated error between Q3 and Q4 had a magnitude of 0.41. Additionally, no remaining modification index suggested a change in Χ^2^ greater than 10.

Finally, to check if any observation had a major influence on the CFA model, we ran a case-level influence analysis using DFBETAs and generalized Cook’s distance (Pek and MacCallum, 2011). The largest change in any loading was 0.065, well below the threshold ( $2/\sqrt{n}$ = 0.166). Also, even the largest Cook’s distance (0.001) was below the cutoff (4/*n* = 0.027). This shows that no single observation had a meaningful impact on the model estimates. As shown in Table 4, all items exhibited salient standardized factor loadings and exceeded 0.65, which is consistent with prior validation of the scale. Model-based reliability and convergent validity were adequate (AVE = 0.687; CR = 0.936).

**Table 4:** CFA item factor loadings.

Item	Factor loading	SE	R^2^
Q1	0.684	0.053	0.468
Q2	0.791	0.038	0.626
Q3	0.848	0.031	0.720
Q4	0.796	0.043	0.633
Q5	0.789	0.055	0.623
Q6	0.901	0.019	0.813
Q7	0.924	0.017	0.854

MLR estimation was used; SE: Standard error.

Exploratory MG-CFA supported the equivalence of the ES-PFSS across gender (Table 5). The configural model showed acceptable overall fit, although its RMSEA was above conventional cutoffs, other fit indices (CFI and SRMR) indicated good model fit, and RMSEA is known to be sensitive to model complexity and degrees of freedom in small or multigroup CFA models. Imposing equality constraints on factor loadings (metric invariance) and on factor loadings and intercepts (scalar invariance) did not significantly worsen model fit, as indicated by non-significant chi-square difference tests and minimal changes in fit indices (ΔCFI ≤ 0.01). Strict invariance constraints similarly did not lead to a meaningful deterioration in model fit.

**Table 5:** Exploratory measurement invariance across gender.

Model	χ^2^	df	CFI	RMSEA	SRMR
Configural	51.11	26	0.968	0.121	0.030
Metric	57.37	32	0.969	0.107	0.051
Scalar	63.80	38	0.969	0.099	0.056
Strict	65.41	45	0.974	0.082	0.058

MLR estimation was used. Reported χ^2^ and fit indices correspond to their robust versions.

The correlated residual between Q3 and Q4 was retained across groups but was not constrained to equality, as constraining this parameter significantly worsened model fit. When freely estimated, the residual covariance was considerably different between women (*r* = 0.26) and men (*r* = 0.62), indicating group differences in the magnitude of this wording-related effect. Overall, results provide exploratory evidence for at least metric and scalar invariance across gender, supporting the comparability of ES-PFSS scores between men and women.

### 3.3 Criterion validity

Regarding criterion validity, Figure 2 shows Pearson’s correlation coefficients calculated between ES-PFSS scores and psychological health and quality of life scales. All of them were statistically significant (*p* < 0.05). As expected, the ES-PFSS test scores positively correlate moderately with DASS-21 Stress, Depression, and Anxiety subscales, as well as with AAQ-II. Complementary, it shows a low negative correlation with the SWLS scores. Also, the average ES-PFSS differed by 6.25 points between satisfied athletes and those who were not (higher scores correspond to athletes who were not satisfied), *t* (123) = 3.625, *p* < 0.001. This implies a Cohen’s *d* of 0.677, and a rank biserial correlation was 0.369.

## 4 Discussion

The general objective of this study was to translate and assess the psychometric properties of the PFSS in Spanish (ES-PFSS) for athletes and their sports context, with the aim of evaluating its adaptability within a Spanish-speaking population. Viewed collectively, these findings suggest that the ES-PFSS demonstrates adequate psychometric performance and supports its adaptability, appropriateness, and applicability within the Spanish sport context. Notwithstanding, adaptation is an ongoing process, where future research and further incorporation and addition of qualitative methods could help explore how athletes interpret and respond to the scale items within their cultural context. The specific objectives included studying the psychometric qualities of the ES-PFSS scale and demonstrating whether they are significant and adaptable to the Spanish context. This study consisted of evaluating its reliability (internal consistency and temporal stability), construct validity, and criterion validity. The ES-PFSS scale presents excellent internal consistency, but moderate test–retest reliability; the construct validity and criterion validity evidence were solid.

In the reliability study, the ES-PFSS scale shows high internal consistency. Test–retest reliability over 5 weeks was low, and the wide 95% confidence interval indicates substantial uncertainty due to the small retest subsample. Importantly, no statistically significant mean-level change between administrations was detected, which reduces concerns about a systematic shift in scores over time. However, individual-level stability remained limited.

Regarding construct validity, factorial studies were carried out, where only one factor was confirmed. These results correspond with those of Johles et al. (2020) and Badinlou et al. (2022), who examined the one-dimensionality of the scale adapted to the sports context in elite athletes. Therefore, the psychometric scale is unidimensional, and it functions as a tool with potential for the measurement of psychological flexibility in athletes. Also, exploratory analyses indicated that ES-PFSS exhibits at least metric and scalar invariance across gender, suggesting that psychological inflexibility in sport is measured in a comparable way for men and women. This finding supports the interpretation of gender differences in associations involving the ES-PFSS and (given appropriate caution) comparisons of the latent averages.

A significant residual covariance (via modification indices) was identified between two items (Q3 and Q4), where it is inferred that the similarity of the statements may have influenced this variation. Statement Q3 (“When I compete, my thoughts weaken/impair my performance”) and Q4 (“When I compete, my feelings weaken/impair my performance”) only differ in that the first one mentions thoughts and the fourth one mentions emotions. On the other hand, the possibility of inferring and evaluating the degree of attention devoted to completing the questionnaire emerges. Although the statements generate confusion due to their similarity, it is pertinent to question the concentration given by the participants to identify the difference in statements and offer reliable results based on their experience.

This residual covariance was not invariant across gender, with a stronger residual association observed in men than in women. It suggests group-specific wording effects, whereby the conceptual overlap between cognitive and affective experiences during competition may vary in magnitude across groups. Such differences may reflect variability in emotion differentiation or in how cognitive and affective experiences are conceptualized during competition (Kashdan et al., 2015), rather than differences in the underlying construct itself, and do not compromise the overall factorial equivalence of the scale. Because of the modest size of the female group (*n* = 52), invariance results should be interpreted cautiously and warrant replication in larger and more balanced samples.

Regarding the first hypothesis, the ES-PFSS proves to be consistent with previous studies in claiming significant positive correlations with the AAQ-II. Therefore, it can be inferred that the ES-PFSS and the AAQ-II measure the same construct, with the former managing to fit the sports context. In line with this, psychological flexibility has been linked to various psychopathological symptoms such as anxiety, stress, depression, and sleep disorders, showing this association even in the sports environment (Bardeen et al., 2013; Kato, 2016). Furthermore, the results show a strong correlation between the ES-PFSS and the stress subscale of DASS-21, while the correlations of anxiety and depression are moderate. This suggests that the negative impact of stress, agitation, and difficulty in managing demands is prominent. Also, ES-PFSS correlates negatively with quality of life (measured by SWLS). Overall, these results are aligned with previous research (Badinlou et al., 2022; Johles et al., 2020; Ruiz, 2010).

Regarding the second hypothesis, a significant mean difference and a low-to-moderate point-biserial correlation were observed between the ES-PFSS and athletic perceived performance measured with the modified Borg CR100 scale. Thus, it is argued that those participants who have low scores on the ES-PFSS (indicating greater psychological flexibility) scale also express feeling good about their athletic outcome and performance. The results are consistent with previous studies, where they established that psychological flexibility is related to preparation prior to the match, performance in competition or sports activity, and the rehabilitation process, by accepting unpleasant thoughts and emotions (Bühlmayer et al., 2017; Gardner and Moore, 2004; Johles et al., 2020).

One relevant limitación of the present study is that, although we followed a structured translation procedure (forward translation and back-translation by experts in clinical psychology), we did not conduct cognitive debriefing interviews or focus groups to determine how athletes interpreted and responded to items. Such methods provide valuable validity evidence based on response processes and they can help identify culture or context-specific interpretations that may not be detected through psychometric analyses alone (Viladrich et al., 2011; Latinjak et al., 2016). Future research should incorporate these qualitative approaches and test cross-national equivalence in other Spanish-speaking contexts.

A second limitation is related to the adaptation of the Borg CR100 scale. Specifically, the original 0–100 response format was modified to a dichotomous subjective scale (Yes/No) to assess the level of satisfaction with the athlete’s last or recent competition. This decision was driven by practical constraints related to participant access and the absence of systematic collaboration with coaches, which limited the feasibility of collecting repeated and fine-grained measurements. Previous studies using the Borg CR100 scale have typically employed a continuous response format ranging from 0 to 100 and have obtained ratings from both athletes and coaches at multiple time points across different phases of the sports process (preparation, competition, recovery). Thus, the Borg CR100 scale was used in a modified form, which requires other statistical analyses, such as the biserial point correlation, since it has metric and dichotomous (binary) variables. Regarding sample access, we consider the limitations of access to participants who are at the professional and world elite level for the study.

Third, given that the sampling method was by convenience, the generalizability of the study is limited. Concerning the sample, there are only 14.4% professional athletes, 29.452% semi-professional athletes, and 56.164% amateur athletes. Being mostly a sample that analyzes amateur participants, this is detrimental to the ecological validity of the study, compared to previous studies that had a semi-professional and professional sample. With the sports practiced by the participants, the sample indeed presented heterogeneity in sports; however, there is a greater prevalence of individual sports than of team sports. Fourth, it is considered a test–retest study that does not yield significant results, mainly due to the sample size of the retest study (n = 13). The low participation in the retest influences the reliability of the intraclass correlation (ICC). By considering a small sample representing 8.9% of the initial participants, the stability of the measurements is considered less represented, affecting the accuracy of the ICC. Finally, as mentioned by Badinlou et al. (2022), the ES-PFSS measures psychological flexibility as a general construct. It is known that such a construct is multifaceted, thus requiring further elaboration of other instruments in a specific sports context.

This study presents several strengths. Firstly, it features a robust factor structure derived from a sample that includes a diverse age range across various sports, with a significant proportion of professional and semi-professional athletes. Additionally, psychological flexibility is examined within a context different from that of elite and sub-elite athletes, such as Swedish and Persian populations.

Regarding future lines of research, Acceptance and Commitment Therapy (ACT) may employ this scale as a measure of performance in the sports context. The study has detailed correlations of interest that deserve further investigation, mainly by continuing to evaluate variables such as employability outside the sports context, sport contractibility, type of sports (individual versus team), sport modalities, age of participants, and their relationship with psychological flexibility in sport. The present study considers that there are factors that influence these Pearson correlations between depression, anxiety and stress, and psychological flexibility in athletes; however, future lines of research are needed to explore further how these variables influence.

As mentioned before, it is pertinent to have a greater number of participants in the retest–test study to provide more significant data concerning the reliability of the study. On the other hand, as mentioned in previous studies (Badinlou et al., 2022; Johles et al., 2020), there is a need for further research, particularly longitudinal studies, to evaluate fluctuations in psychological flexibility among athletes. There is also a need for further instrument development at the sport level, using the ES-PFSS as a starting point and inspiration in the development of other tools that explore and delve deeper into psychological flexibility in athletes. In this line, the development of different instruments can also contribute to the comparison of results and to evaluating the validity of the scale.

Given the statistically significant results of this study, it is pertinent to explore the effectiveness of Acceptance and Commitment Therapy in athletes within their sporting context, utilizing the ES-PFSS scale as a tool to evaluate psychological flexibility. Moreover, the ES-PFSS scale may provide valuable information for psychology professionals to support more effective intervention and assessment of athletes. Although the present study provides evidence for a Spanish-language version of the instrument, the validation was conducted in a sample from Spain. Linguistic and cultural variations across other Spanish-speaking countries may influence how psychological flexibility is expressed in sport. Therefore, further studies should examine the cross-national validity and invariance of the ES-PFSS in other Spanish-speaking contexts. Finally, future research may benefit from cultural praxis perspectives (Ryba et al., 2024) to guide cross-cultural work in sport psychology and to better capture contextually based meaning of psychological flexibility across sport and cultural settings.

## Data Availability

The raw data supporting the conclusions of this article will be made available by the authors, without undue reservation.
